# Efficient production of a self-amplifying RNA vaccine sequence with the single subunit RNA polymerase from *Klebsiella* phage 32 utilizing the native AU initiating dinucleotide

**DOI:** 10.1261/rna.081020.126

**Published:** 2026-10

**Authors:** Marcus A. Price, Maryia Trubitsyna, Jane E. Paget, Benjamin W. Baker, Frances Turner, Susan J. Rosser

**Affiliations:** School of Biological Sciences, Centre for Engineering Biology, University of Edinburgh, Edinburgh EH9 3BF, United Kingdom

**Keywords:** RNA polymerase, saRNA, mRNA production, Ψ-UTP

## Abstract

RNA polymerase (RNAP) is the enzyme that produces RNA for an increasingly large variety of uses. The need for efficient industrial production of high quantities of mRNA was exemplified during the SARS-CoV-2 pandemic. While the RNAP from the T7 bacteriophage has been the gold standard for some time, it has certain limitations impeding efficient manufacturing of all RNA types, including production of long transcript self-amplifying RNA (saRNA). Here we present an alternative to T7 RNAP, identified from the *Klebsiella* bacteriophage 32 (KP32). We show KP32 RNAP to efficiently produce RNA of varying lengths, with a wide temperature, pH and salt tolerance range. We show KP32 RNAP to be capable of efficient production of an 11,551 nt self-amplifying RNA with its critical native initiating dinucleotide, AU. This represented a 9.3-fold improvement in production levels, relative to T7 RNAP.

## INTRODUCTION

In vitro transcription (IVT) of RNA by single subunit DNA-dependent RNA polymerase (RNAP) is a vital step for the production of RNAs for therapeutics and other purposes. Most notably, this was used to great effect during the SARS-CoV-2 pandemic by Moderna, BioNTech and Pfizer to produce their mRNA vaccines, enabling the in vivo expression of the spike protein and providing the patient with immunity against this coronavirus. This success has led to a rapid increase in mRNA-based therapeutics progressing to clinical trials for a myriad of applications (Webb et al. 2022).

Self-amplifying RNA (saRNA) vaccines, as the name suggests, are capable of generating mRNA for a protein of interest (POI) within cells, meaning a small dose of the full saRNA can lead to a high therapeutic dose of the POI mRNA. India and Japan were the initial countries to approve saRNA vaccines for use in humans against SARS-CoV-2 in 2022 and 2023, respectively (Silva-Pilipich et al. 2024). Within saRNAs, the RNA sequence is generally based on the Trinidad donkey Venezuelan Equine Encephalitis Virus (VEEV) alphavirus genome, with the viral structural protein sequences replaced with that of a POI. Critical to this is the accurate transcription of the sequence responsible for in vivo mRNA amplification. In particular, it has been noted that the native AU initiating dinucleotide, highly conserved in alphaviruses, is essential for efficient amplification of the POI mRNA (Hyde et al. 2015).

However, the most widely used RNAP for the production of saRNAs, and other in vitro transcribed RNA products, remains the long studied single subunit RNAP from the *Escherichia coli* bacteriophage T7 (T7 RNAP). While T7 RNAP can yield high amounts of RNA from an IVT reaction, it can struggle in the areas of long transcript production, abortive initiation, utilization of initiating nucleotides other than guanine, as well as the wild-type (WT) RNAP having limitations in incorporation of modified rNTPs. Significant literature exists around the use and modification of T7 RNAP (Padilla and Sousa 2002; Borkotoky and Murali 2018), while other single subunit RNAPs have also been identified from alternative bacteriophages, such as T3 (Leary et al. 1991), Syn5 (Zhu et al. 2013, 2014, 2015), SP6 (Butler and Chamberlin 1982) and K11 (Han et al. 1999; Kyung et al. 2002) RNAPs. However, the other identified RNAPs, and modifications of T7 RNAP, have as yet not solved all these issues. Syn5, for example, was developed with long transcript production in mind due to the distance between promoter sequences within its bacteriophage genome (Zhu et al. 2013, 2014, 2015), while KP34 RNAP was selected partially due to low sequence identity to T7 RNAP, and was also found to operate efficiently at lower temperatures than T7 RNAP (Lu et al. 2019). However, neither of these were subsequently used for the transcription of RNA for vaccines against SARS-CoV-2, nor are they widely used in the laboratory to the best of our knowledge. There as such remains a need for new RNAPs capable of producing high quantities of high-quality RNA of various lengths, while also remaining simple to purify and use across a broad range of conditions.

*Autographiviridae* are bacteriophages, such as T7, which encode their own RNAP for production of RNA when infecting a host (Lavigne et al. 2008). They represent a wealth of genetic information from which RNAPs with novel characteristics can be sourced. Having a library of RNAPs available during the development of RNA molecules for therapeutic or industrial applications is important. Such a toolkit could broaden the capabilities of RNA molecules by, for example, enabling utilization when the initiating nucleotide is not guanine; increasing the robustness of transcription-based biosensors in real world environments, where environmental contaminants can interfere with pH, and temperatures cannot be easily controlled (Jung et al. 2020); strengthening cell free production systems to produce maximal output of a target protein or molecule; or during the development of genetic circuits to increase the level of multiplexing possible between orthogonal genetic switches (Temme et al. 2012).

This study identifies and characterizes the RNAP from *Klebsiella* phage 32 (KP32), as well as its cognate promoter sequences. Within the 4195 *Autographiviridae* genomes screened, 127 had been derived from a *Klebsiella* bacterial host. The putative RNAPs from *Klebsiella* bacteriophages carry a conserved 20 amino acid (aa) motif within the N-terminal domain that is not found in T7 RNAP. We identified 112 protein sequences with 100% coverage ranging from 100% to 40% identity relative to KP32 RNAP. We initially characterize KP32 RNAP and subsequently investigate the beneficial attributes this 20 aa motif confers to KP32 RNAP. We lastly show that KP32 RNAP can transcribe high quantities of RNA of various lengths, including the clinically relevant Pfizer/BioNTech mRNA vaccine, and an saRNA vaccine candidate against SARS-CoV-2 with its critical initiating AU dinucleotide. KP32 RNAP produced greater total RNA output, relative to T7 RNAP, from this 4282 nt mRNA vaccine sequence (up to 2.6-fold), as well as the 11,551 nt saRNA sequence (up to 9.3-fold) with the AU initiating dinucleotide. This was found to be true with both standard rNTPs, as well as when replacing UTP with N^1^-methylpseudo-UTP (m^1^Ψ-UTP), as was used by Pfizer/BioNTech within their vaccine to enable a greater therapeutic effect by reducing the cellular response to the foreign RNA (Karikó et al. 2008; Andries et al. 2015; World Health Organization 2020).

## RESULTS

### In silico T7-like RNAP selection and structural analysis

A BLAST search of the T7 RNAP protein sequence was performed against the NCBI genome database to identify potentially interesting *Autographiviridae* RNAP candidates. This method led to the identification of *Klebsiella* phage KP32 RNAP (YP_003347522.1) which was considered to have properties, such as relative similarity of the specificity loop to that of T7 RNAP, which would be advantageous for a characterization target, requiring parallel identification of a functional promoter sequence. When aligned to the T7 RNAP protein sequence (Supplemental Fig. 1), KP32 RNAP shares 72.85% position-by-position identity with the industry standard polymerase, while differing significantly to another *Klebsiella* phage RNAP, KP34 RNAP, with 23.11% identity. This was considered to improve the likelihood of identifying promoter sequences for KP32 RNAP, based on homology with the T7 RNAP promoter, and more generally ease the initial use of this new RNAP in known reaction conditions that have been optimized for T7 RNAP.

However, despite this general similarity, KP32 RNAP has significantly less sequence identity (59.38%) within the 32 aa region that aligns to the specificity loop in T7 RNAP. This specificity loop is known to recognize the T7 promoter during initiation (Cheetham and Steitz 2000), and it was thus predicted that this difference may be sufficient to confer promoter orthogonality between KP32 and T7 RNAPs (Temme et al. 2012). Furthermore, it was observed in the alignment of the N-terminal domains that a 20 aa motif of KP32 did not appear to have any counterpart within T7 RNAP. Further investigation demonstrated that this 20 aa motif was well conserved among other *Klebsiella* phage RNAPs, despite its total absence from the T7 RNAP sequence. One hundred and twelve other *Klebsiella* phage RNAP sequences were identified, all of which contained an equivalent 20 aa motif, with sequence identities ranging from 40% to 100% in comparison to KP32 RNAP. This evidence of evolutionary maintenance of the 20 aa motif suggested that it may confer benefits to transcription that are absent in T7 RNAP.

To further expand upon this investigation, the KP32 protein sequence was run through the AlphaFold 3 server pipeline (Abramson et al. 2024) to generate a predicted structure for the RNAP. Due to the fundamental underpinning of the AlphaFold workflow on multiple sequence alignments (Jumper et al. 2021) this process led to a regeneration of the results already noted in the initial bioinformatic investigation. The first sequence hit in the MSA used by AlphaFold 3 was T7 RNAP due to the general sequence similarity but among the top 20 hits, only five contained sequences aligning to the 20 aa motif, of which three were other *Klebsiella* phage RNAPs. The predicted structure generated had a high confidence level with an average atomic pLDDT score of 85.92 across the whole protein sequence and despite the significantly lower alignment coverage for the 20 aa motif, its structure was predicted with an average pLDDT score of 74.63 (Fig. 1A).

**FIGURE 1.**
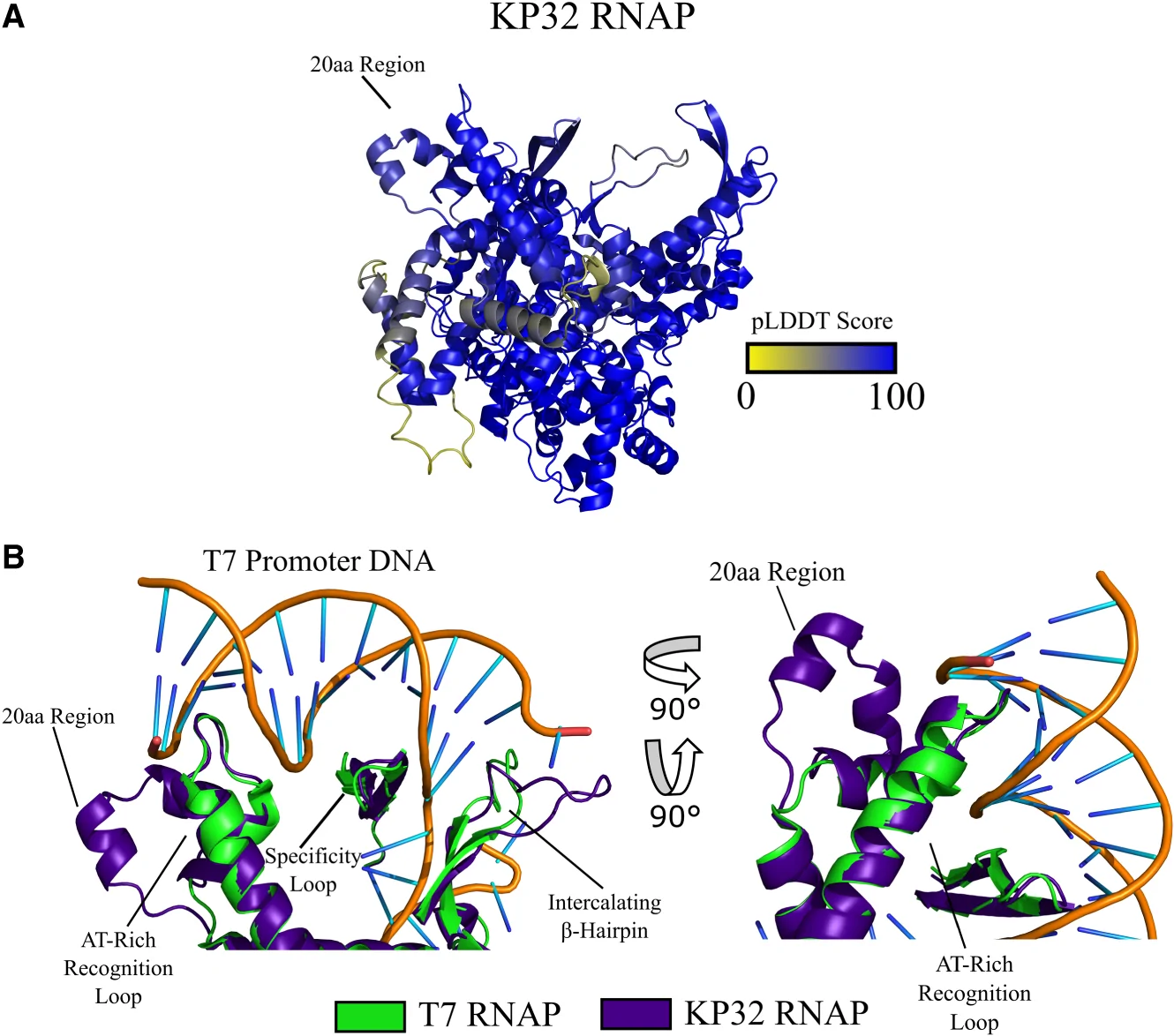
KP32 RNAP predicted structural analysis. (*A*) AlphaFold 3 model of KP32 RNA Polymerase visualized using PyMOL. Structure is colored using a linear yellow to blue spectrum based on atomic pLDDT confidence score reported by AlphaFold. (*B*) Alignment of DNA binding regions in T7 RNAP structure 1QLN (green) and KP32 RNAP AlphaFold model (purple); alignment was performed on whole structure of both proteins but regions other than those proximal to the promoter DNA were hidden for focused analysis of DNA binding domains. Full structure alignment can be found in Supplemental Figure 2.

To aid in analysis of the KP32 RNAP model, a reverse structure search was carried out using FoldSeek (van Kempen et al. 2024) to identify experimental RNAP structures with which it would be informative to align. Unsurprisingly given the sequence similarity and relative wealth of experimental structures available, this process identified the best alignment target to be an x-ray crystallography structure of T7 RNAP. Specifically, the structure highlighted was 1QLN which is of an initiation-elongation complex in which T7 RNAP is bound to its promoter DNA and stalled during the transcription of the nucleotide at the +4 position (Cheetham et al. 1999). Alignment of the model for KP32 RNAP to this T7 RNAP initiation complex showed a generally good match with a global average RMSD score of 2.989 (882 residues to 882) between the two structures and a refined RMSD of 0.490 reported by PyMOL following automatic trimming of outlier regions (629 residues to 629).

Analysis of this alignment was focused on the DNA binding regions upstream of the active transcription site which are well-defined in this model due to the inclusion of the T7 promoter in the crystal structure. Cheetham et al. designated three key DNA binding regions of T7 RNAP from this structure; an intercalating β-hairpin, the specificity loop and an AT-rich recognition loop (Fig. 1B). The AlphaFold structure for KP32 RNAP aligns well to 1QLN in these regions with those same three DNA-binding features clearly visible (full alignment in Supplemental Fig. 2). However, it is also clear from this alignment that the 20 aa motif in KP32 forms two short helices, proximal to the AT-rich recognition loop, that are not present in the T7 RNAP structure. These two helices formed by the 20 aa motif are positioned close to the DNA backbone in this alignment and it is therefore considered possible that they provide an additional DNA contact during the initiation phase of transcription. Given the predictive nature of both the AlphaFold structure and its alignment to the 1QLN T7 structure, this was not alone considered sufficient evidence to define the 20 aa motif as an additional DNA-binding element in KP32 and other *Klebsiella* phage RNAPs. However, this was a highly intriguing structural property which was postulated to confer additional benefits to transcriptional activity of KP32 RNAP and warranted characterization of the polymerase's in vitro transcription activity.

### KP32 RNAP promoter identification

To identify the cognate promoters for KP32 RNAP, the intergenic regions within the KP32 phage genome were searched for sequences which aligned to the T7 promoter. Extrapolation from the T7 promoter would presume that the KP32 promoter is 18 bp inclusive of a guanine as the transcriptional start site. Ten sense-strand, intergenic sites were revealed with five mismatches to the T7 promoter sequence. Three candidate promoters were selected due to repetition within the genome (5′-AATTAGGGCACACTATAG—repeated five times; 5′-AATTAGGACCCACTATAG—repeated three times), or positioning upstream of a well annotated gene encoding a DNA ligase (5′-AATTAGGACACACTATAG). To ensure that the tested sequences would encompass the entire promoter, three aligned putative promoters were chosen along with a further six bases upstream of the putative 5′ end, as well as 10–11 bases downstream from the putative transcriptional start site (TSS). These three promoters were designated P_*KP32_1*_ (5′-TCAGGCAATTAGGGCACACTATAGGGACCCGCTC—the underlined base represents the putative TSS), P_*KP32_2*_ (5′-GGAAGTAATTAGGACACACTATAGGCAGACTCAAG) and P_*KP32_3*_ (5′-GTTATTAATTAGGACCCACTATAGGAGACACTAAA).

### In vivo promoter validation

To test the activity of KP32 RNAP on the three putative promoters selected, the KP32 WT RNAP gene was placed under the control of an arabinose inducible promoter with a weak ribosomal binding site (RBS) to reduce potential toxicity (Fig. 2A). The resulting low-copy plasmid was co-transformed into *E. coli* TOP10 cells with a second plasmid containing one of the candidate KP32 RNAP promoters from which the GFP fluorescent reporter can be expressed, utilizing an alternative weak RBS. As a control, T7 RNAP was tested alongside KP32 RNAP using P_T7_. This analysis qualitatively identified P_*KP32_2*_ as the best promoter for KP32 RNAP (Fig. 2B). Replicate reaction plate images and semi-quantitative analysis can be found in Supplemental Figure 3. Further quantifiable validation of P_*KP32_2*_ was obtained by incubating liquid cultures either in the presence of 0.2% glucose to repress the expression of GFP, or 0.2% arabinose to induce expression of the RNAP, prior to fluorescence analysis. The region of P_*KP32_2*_ downstream from the putative TSS was removed, and the effect of the removal of the region upstream region of the core 18 bp promoter with homology with P_T7_ was analyzed (Fig. 2C). It was revealed that while the core 18 bp promoter was capable of enabling GFP expression, the 24 bp sequence (GGAAGTAATTAGGACACACTATAG) produced a greater average level of GFP (Fig. 2C), and, while not statistically significantly different, was taken forward for use in further studies and hereafter designated as P_*KP32*_.

**FIGURE 2.**
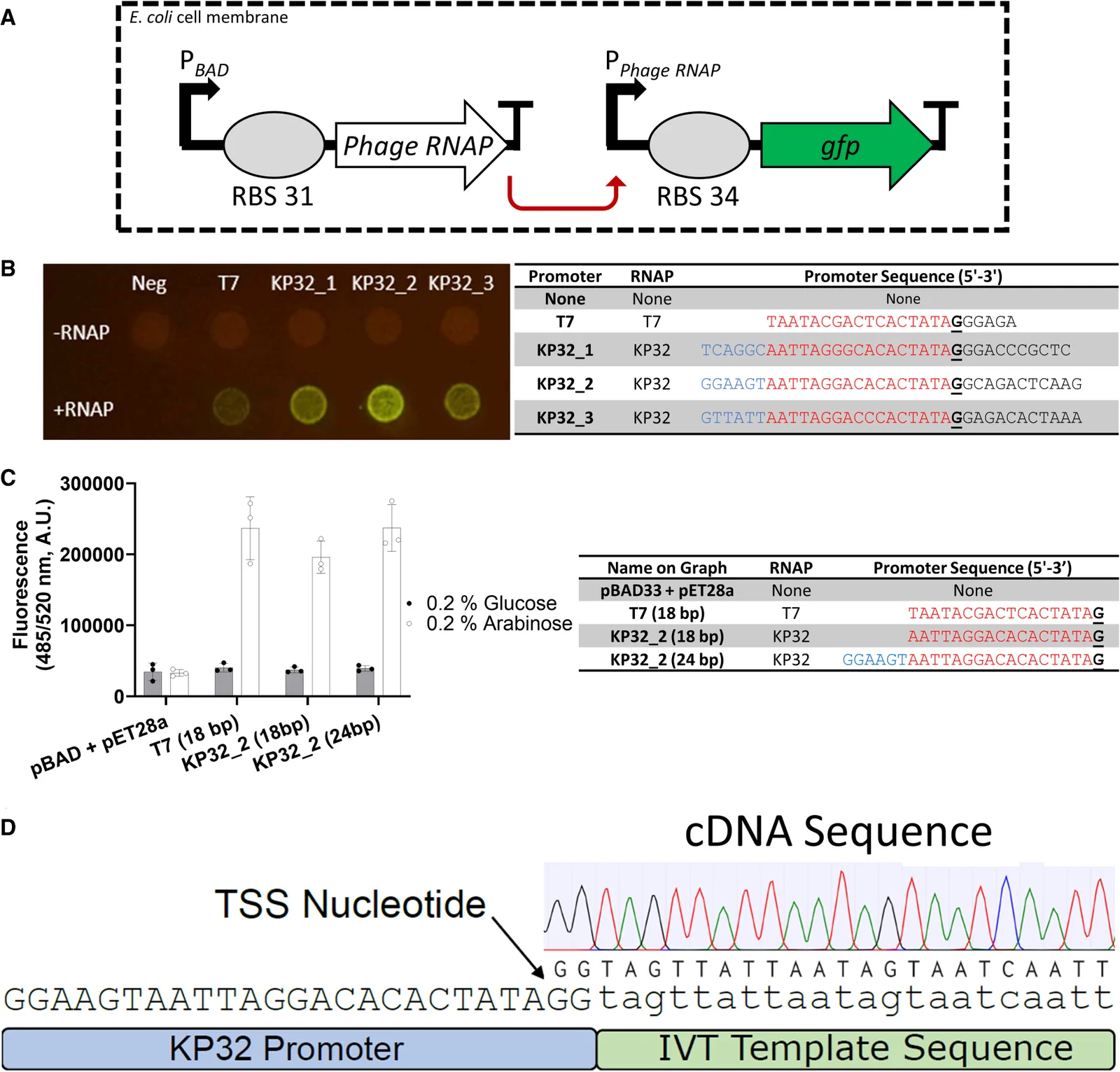
Candidate promoter analysis for KP32 RNAP. (*A*) Schematic diagram describing the semi-quantitative assays performed using a two-plasmid system where *gfp* expression is placed under the control of a putative phage RNAP promoter. Transcription from this promoter only occurs if the second plasmid expresses a cognate phage polymerase under arabinose inducible P_*BAD*_. (*B*) Florescence was observed by placing cells expressing GFP under a blue light box (470/530). Promoter sequences were tested with (+RNAP) and without (−RNAP) their cognate RNA polymerase. An empty pBAD33 plasmid was used in – RNAP strains. The negative control (Neg) carries the empty pBAD33 and pET28a vectors. The image is a representative image of triplicate plates—images of all plates can be found in Supplemental Figure 3. (*C*) Fluorescence levels of cultures following 4 h of growth with either 0.2% glucose as a negative control to repress expression of the RNAP, or 0.2% arabinose to induce expression of the RNAP. Promoters tested analyze the 24 bp and minimal version of P_*KP32_2*_ against the T7 RNAP system. The average and individual readings, alongside error bars representing the standard deviation, of three replicates is shown. (*D*) Template switching process to determine the TSS. The final Sanger sequencing chromatogram is shown alongside a schematic of the relevant genetic elements.

To confirm the TSS, a template switching reverse transcription approach was taken. This utilizes a reverse transcriptase that adds nontemplated DNA bases to the end of the cDNA it generates which can then bind to a template-switching oligonucleotide (TSO). This in turn allows for a barcode sequence to be introduced into the 3′ end of cDNA and for subsequent PCR amplification to preserve the TSS information for Sanger sequencing. The result of this process is shown in Figure 2D and validated that the underlined base in Figure 2C was indeed the TSS.

### Promoter orthogonality of T7 RNAP and KP32 RNAP

KP32 WT RNAP and T7 RNAP were purified and compared by IVT reactions at equal concentrations. As discussed, in silico analysis showed the main structural point of difference between KP32 RNAP and T7 RNAPs to be a 20 aa (108–127) motif in the N-terminal domain of KP32 RNAP. However, the function of this motif has not been determined, and within *Klebsiella* phages the region is highly conserved with only three residues showing variation >5% across the family. This suggests that the 20 aa motif could play an important role within the *Klebsiella* phage RNAP functionality. Thus, to investigate the function of the 20 aa we constructed a deletion mutant (KP32 Δ20 aa RNAP), purified this variant and included it in comparison assays to KP32 WT RNAP and T7 RNAP (Fig. 3).

**FIGURE 3.**
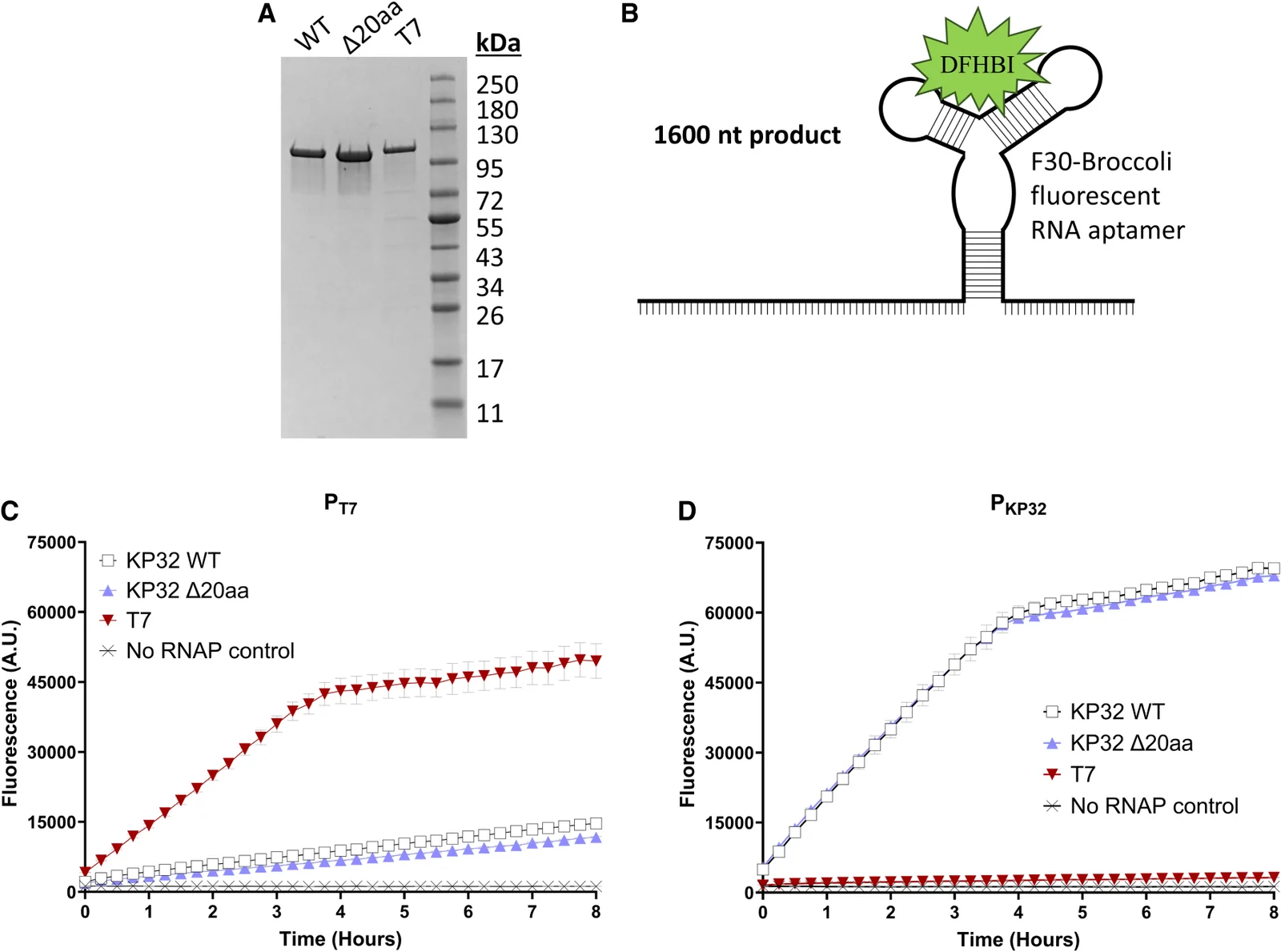
Purification and orthogonality of T7, KP32 WT and KP32 Δ20aa RNAPs. (*A*) SDS-PAGE analysis of purified KP32 WT RNAP (103 kDa), KP32 Δ20 aa RNAP (101 kDa) and T7 RNAP (102 kDa). (*B*) Schematic representation of the transcribed 1600 nt RNA product. F30-Broccoli is produced and interacts with DFHBI to elicit a fluorescent response. (*C*) Fluorescent responses throughout IVT reactions with normalized RNAP concentrations and the 1600 bp template containing P_T7_. (*D*) Fluorescent responses throughout IVT reactions with normalized RNAP concentrations and the 1600 bp template containing the P_*KP32*_. iPPase was not included in these reactions. All reactions were performed in triplicate, and error bars represent the standard deviation.

As a reporter for efficient transcription, the fluorescent RNA aptamer, broccoli, was utilized. Fluorescent RNA aptamers such as broccoli are a useful tool to investigate RNAP output (Kartje et al. 2021). The fluorogen 3,5-difluoro-4-hydroxybenzylidene imidazolinone (DFHBI), when bound to the broccoli aptamer, mimics the output of the green fluorescent protein (GFP). Thus, this tool can be utilized to investigate production rates in real time. Further, folding of the broccoli aptamer can be improved through the use of the F30 scaffold without inhibiting fluorescence of the aptamer:fluorogen duplex (Filonov et al. 2015). Lastly, F30-Broccoli can be added to the 3′ end of RNA transcripts while maintaining its fluorescence output, making it an ideal tool to investigate RNAP activity on transcripts of increased length.

A 1600 nt transcript was utilized to investigate RNAP activity with the F30-Broccoli aptamer at the 3′ end indicating transcription of the full DNA template (sequence available in the Supplemental Material) prepared by PCR (Fig. 3B). As broccoli fluorescence is known to be more stable at 25°C rather than 37°C (the optimal transcription temperature for T7 RNAP), IVT reactions tracking broccoli production were set up at 25°C (Fig. 3C,D). A clear increase in transcriptional efficiency was observed with KP32 WT RNAP in comparison to T7 RNAP at 25°C. These results indicated that, at least at 25°C, KP32 WT RNAP and KP32 Δ20 aa RNAP were capable of producing more RNA at a faster rate in an IVT reaction in comparison to T7 RNAP, purified in our hands. Furthermore, it was found that T7 RNAP had no significant activity above background on templates beginning with P_KP32_. KP32 WT RNAP and KP32 Δ20 aa RNAP were found to have a low level of activity when utilizing P_T7_; however, this was found to be 6.7- and 8.7-fold less than when utilizing P_KP32_, after 4 h incubation, for KP32 WT RNAP and KP32 Δ20 aa RNAP, respectively.

### Characterization of KP32 RNAPs in comparison to T7 RNAP

To characterize KP32 RNAPs, the purified WT and Δ20 aa enzymes were subjected to temperature and pH optima screens, as well as analyzing their tolerance to NaCl, inorganic pyrophosphate and various DNA template concentrations, in comparison to T7 RNAP.

#### Temperature optima screen

To analyze the optimum temperature for activity of KP32 WT, KP32 Δ20 aa and T7 RNAPs, IVT reactions were set up and analyzed at 21°C, 25°C, 30°C and 37°C. The pH of these assays was controlled to pH 7.9 as this is the standard pH in reactions buffers supplied with commercial T7 RNAPs. Following DNase treatment to remove the IVT template, and utilization of an RNA clean-up kit to remove other IVT reaction components, RNA concentrations were measured by nanodrop (Fig. 4A). Unpaired *t*-tests with Welch's correction were performed to analyze these results and can be viewed in Supplemental Table 1. The results indicate that under the tested conditions, KP32 WT RNAP is most active at 30°C, while retaining strong activity rates at 21°C and 25°C. Other than 37°C, KP32 WT RNAP outperformed T7 RNAP total RNA yield at all tested temperatures. KP32 WT RNAP outperformed KP32 Δ20 aa RNAP at all temperatures, indicating the 20 aa motif affects the RNAPs rate of processivity. Agarose gel images of the purified RNA products can be viewed in Supplemental Figure 4. These show that throughout all reactions, the primary RNA product was the 1600 nt product. A secondary band can be observed in some reactions, in particular with T7 RNAP. As this band was approximately double the size of the target product, and the loaded RNA had been heat treated to relax the RNA, we hypothesized this to be dsRNA. Further, the strong interactions associated with the F30 stabilization region added to broccoli could act as a priming region of the known RNA dependent RNAP activity of T7 RNAP (Wu et al. 2021). The optimal 30°C reactions for KP32 WT and Δ20 aa RNAPs, and the 37°C reactions for T7 RNAP were investigated for dsRNA presence by spot assay (Supplemental Fig. 5). This spot assay shows dsRNA to be generated in all reactions with KP32 WT and Δ20 aa RNAPs producing no more dsRNA than T7 RNAP. The aim of this dsRNA analysis was to show a comparison of dsRNA formation between KP32 WT and Δ20 aa RNAPs against T7 RNAP. Quantification is difficult as the 1600 nt template represents a far greater region of potential dsRNA upon which the J2 antibody, requiring only 14 nt of dsRNA to bind. Further studies should be undertaken for an exact quantification of dsRNA levels.

**FIGURE 4.**
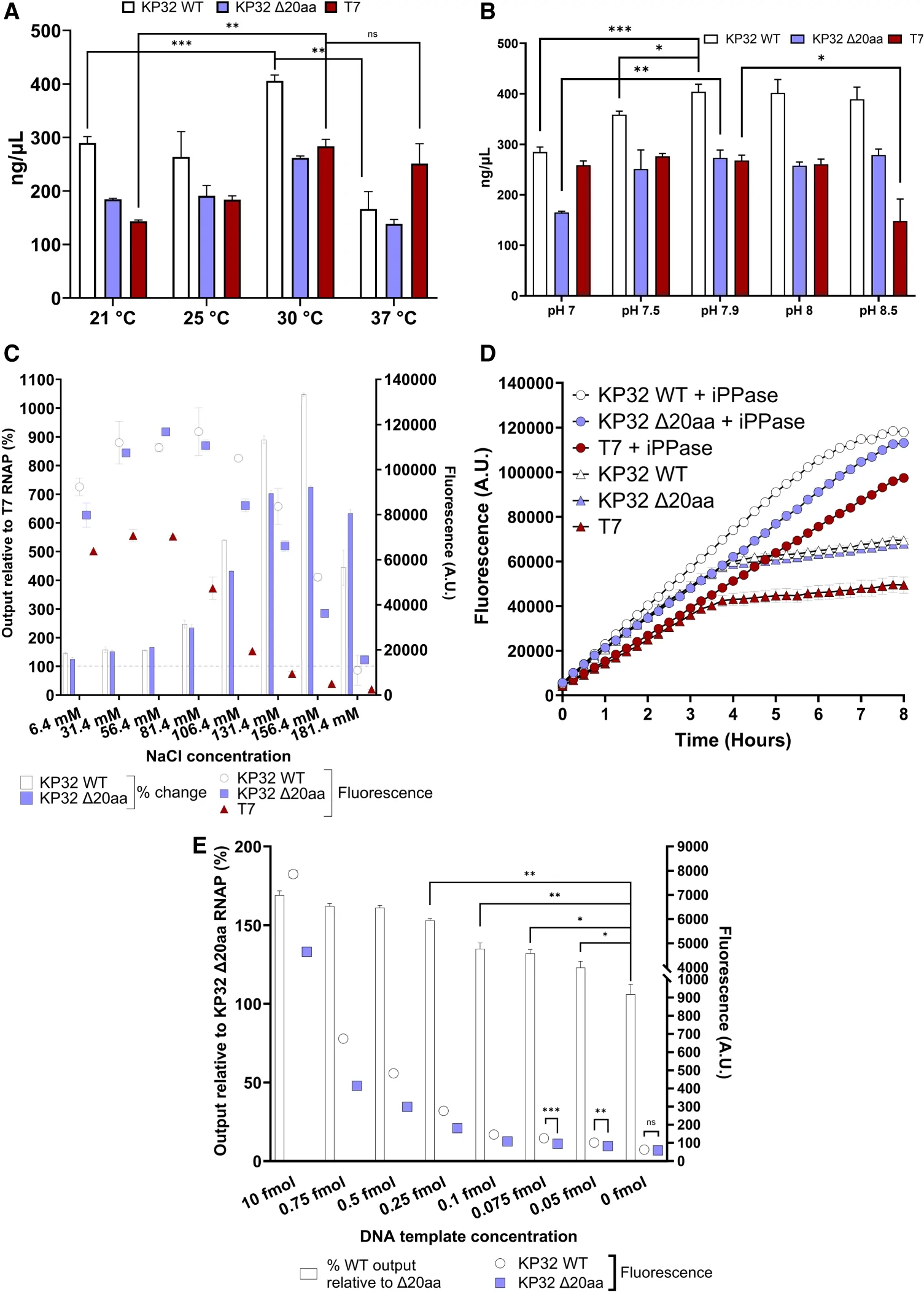
Characterization of KP32 WT, KP32 Δ20 aa and T7 RNAPs. (*A*) Optimal reaction temperature assay. Total RNA output following 2 h IVT reactions at varying temperatures. Selected *P*-value summaries are shown. Full *P*-value summaries obtained following unpaired *t*-tests with Welch's correction can be found in Supplemental Table 1. (*B*) Optimal pH assay. Total RNA output following 2 h IVT reactions at varying pHs. All reactions were incubated at 30°C. Selected *P*-value summaries are shown. Full *P*-value summaries obtained following unpaired *t*-tests with Welch's correction can be found in Supplemental Table 2. (*C*) Tolerance to NaCl assay. Fluorescence output and output relative to T7 RNAP following 5 h of IVT of a 1600 nt transcript, containing F30-Broccoli, with varying concentrations of NaCl. (*D*) Reaction output analysis with and without iPPase. Time course assay showing fluorescence output following IVT with a 1600 nt transcript, containing F30-Broccoli. (*E*) Utilization of low concentration IVT template DNA. Fluorescence output of KP32 WT and KP32 Δ20 aa RNAPs, and output of KP32 WT RNAP relative to KP32 Δ20 aa RNAP, following 4 h of IVT with varying concentrations of 105 bp DNA F30-Broccoli. In all graphs, *n* = 3 with the mean and standard deviation shown. Where significance levels are indicated, unpaired *t*-tests with Welch's correction were performed. (*) *P* < 0.05, (**) *P* < 0.01, (***) *P* < 0.001.

#### pH optima screen

To analyze the optimum pH for activity of KP32 WT, KP32 Δ20 aa and T7 RNAPs, IVT reactions were set up using 10x concentrated RNAP reaction buffers with altered pHs. All reactions were performed at 30°C as pH is known to be affected by temperature and we did not wish to introduce any unintended variations (Frant 1995). Following DNase treatment to remove the IVT template, and utilization of an RNA clean-up kit to remove other IVT reaction components, RNA concentrations were measured by nanodrop (Fig. 4B). Unpaired *t*-tests with Welch's correction were performed to analyze these results and can be viewed in Supplemental Table 2. The results indicate that the optimum reaction pH for KP32 WT RNAP and KP32 Δ20 aa RNAP does not vary to T7 RNAP (pH 7.9). To ensure the use of 30°C within the reaction condition did not bias our results, a similar assay was performed at the identified optimal temperature for each RNAP. These showed the same pattern of results, and subsequent conclusion, as observed in the exclusively 30°C reactions (Supplemental Fig. 6) These assays also show KP32 WT RNAP as having an increased tolerance to slightly elevated pHs than T7 RNAP. Agarose gel images of the purified RNA products can be viewed in Supplemental Figure 7. Similar to the temperature optima results, these show that throughout all reactions, the primary RNA product was the 1600 nt product while a secondary band can be observed in the T7 RNAP samples.

#### Salt tolerance analysis

T7 RNAP is known to be susceptible to inhibition by monovalent salt ions (Milligan et al. 1987). To determine the tolerance of KP32, KP32 Δ20 aa, and T7 RNAPs to NaCl, reactions were set up with an increasing gradient of NaCl added to the reactions. Reactions were set up using the Certus Flex and ECHO liquid handling equipment in the Edinburgh Genome Foundry. Salt tolerance was investigated at 6.4, 31.4, 56.4, 81.4, 106.4, 131.4, 156.4, and 181.4 mM NaCl—this considers the 6.4 mM NaCl added alongside the RNAP, within the RNAP storage buffer. Fluorescence was tracked from F30-Broccoli production within the 1600 nt IVT transcript. Reactions were performed at 25°C, pH 7.9 as this is the standard reaction buffer used for T7 RNAP. The results after 5 h incubation (Fig. 4C) confirm T7 RNAP does not have a high tolerance to NaCl, with optimal fluorescence output at 31.4 mM NaCl. KP32 WT and KP32 Δ20 aa RNAPs on the other hand exhibit an increased tolerance to NaCl, with optimal fluorescence output at 81.4 mM. Furthermore, KP32 WT and KP32 Δ20 aa RNAPs do not lose activity as drastically as T7 RNAP, with 10.5- and 7.2-fold more fluorescence still detected at 156.4 mM NaCl, respectively. Lastly, KP32 WT RNAP appears to be somewhat more tolerant of NaCl than KP32 Δ20 aa RNAP. At 6.4–81.4 mM NaCl, no significant difference was observed after 5 h. However, at this time point, 106.4 and 156.4 mM NaCl reactions with KP32 WT RNAP show a significantly higher fluorescence output than KP32 Δ20 aa RNAP (*P* = 0.0056 and 0.0026, respectively [unpaired *t*-test with Welch's correction]). Full 12 h time course reaction data can be found in Supplemental Figure 8.

#### Requirement of inorganic pyrophosphase to maximize RNA yield

Inorganic pyrophosphate (iPP) is a by-product of polymerase reactions which is known to inhibit polymerization. As such, inorganic pyrophosphatase (iPPase) is included within IVT reactions to maximize yield. To investigate the requirement of iPPase, the level of RNA production from KP32 WT, KP32 Δ20 aa, and T7 RNAPs was determined in the presence or absence of iPPase. Fluorescence was tracked from F30-Broccoli production within the 1600 nt IVT transcript. The results indicate a reduced need for iPPase by KP32 WT and KP32 Δ20 aa RNAPs, relative to T7 RNAP (Fig. 4D). After 4 h of incubation, reactions without iPPase appear to cease production of F30-Broccoli, while reactions with iPPase continue production. After 4.5 h incubation, KP32 WT RNAP had produced 41.5% more fluorescence than T7 RNAP, when both sets of reactions lack iPPase. One of the primary effects of iPP is to bind free Mg^2+^ ions, forming insoluble Mg_2_PPi. This formation has the effect to inhibit polymerization reactions as the RNAP requires free Mg^2+^ ions as a cofactor (Yang et al. 2006). Yang et al. (2006) postulate that small differences in metal ion binding energy could cause large differences in catalytic efficiency between different polymerases. This could explain the difference in catalytic rate between KP32 WT and KP32 Δ20 aa RNAPs, relative to T7 RNAP, while also representing a potential tolerance to lower free Mg^2+^ ion concentrations and thus a lower requirement for the addition of iPPase as shown here.

#### Effect of IVT template DNA concentration on RNA output

Since the 20 aa motif not found in T7 RNAP is in the N-terminal region of KP32, in the proximity of the promoter -13 to -17 base binding site, and the promoter intercalating hairpin loop (Borkotoky et al. 2017), it was proposed that these 20 residues could be involved in the recruitment of template DNA where the promoter is present. To test this hypothesis, reactions were set up where the total added template of the 105 bp F30-broccoli DNA template was lowered and varied from 10 to 0.05 fmol. The shorter 105 nt transcript (the sequence and PCR primers for which can be found in the Supplemental Material) was used here to increase the sensitivity of the reaction by producing more of the smaller fluorescent RNA aptamer transcript. The results reveal that while KP32 WT RNAP consistently produces a greater fluorescence output than KP32 Δ20 aa RNAP, the relative output does not change when >0.5 fmol template DNA is present (Fig. 4E), indicating saturation of the template DNA. Further, when template DNA is between 0.05 and 0.25 fmol, KP32 WT RNAP loses fluorescence production levels faster than KP32 Δ20 aa RNAP. To ensure the results were statistically significant at the lowest template DNA concentrations, unpaired *t*-tests with Welch's correction were performed for 0.05 fmol (*P* = 0.0015) and 0.075 fmol (*P* = 0.0002) results. The no template DNA was also found to not be a significant difference. While preliminary, this therefore disproves our original hypothesis as, without the 20 aa motif, template recruitment efficiency by KP32 Δ20 aa RNAP slightly increases when template concentration is the rate limiting step. Full 12 h time course reaction data can be found in Supplemental Figure 9.

### SARS-CoV-2 spike protein mRNA production and RNAP fidelity analysis

To investigate the ability for KP32 WT and KP32 Δ20 aa RNAPs to produce longer RNA transcripts, in comparison to T7 RNAP, the 4282 nt sequence for the SARS-CoV-2 mRNA vaccine, produced by BioNTech/Pfizer and publicly released by the WHO (World Health Organization 2020), was synthesized and inserted into a pUC19 plasmid backbone. The plasmid was linearized and IVT reactions were performed at the optimum temperature identified for each RNAP (30°C or 37°C), as well as a 21°C “room temperature” assay. Following a 2 h incubation, reactions were halted with DNase treatment to destroy the template. The concentration of purified RNA was measured by nanodrop following utilization of an RNA clean-up kit to remove other IVT reaction components (Fig. 5A). The results show that with unmodified rNTPs, KP32 WT RNAP outperforms KP32 Δ20 aa RNAP and T7 RNAP at 21°C (1.7- and 2.6-fold, respectively), as well as at optimum reaction temperatures (1.4- and 1.9-fold, respectively).

**FIGURE 5.**
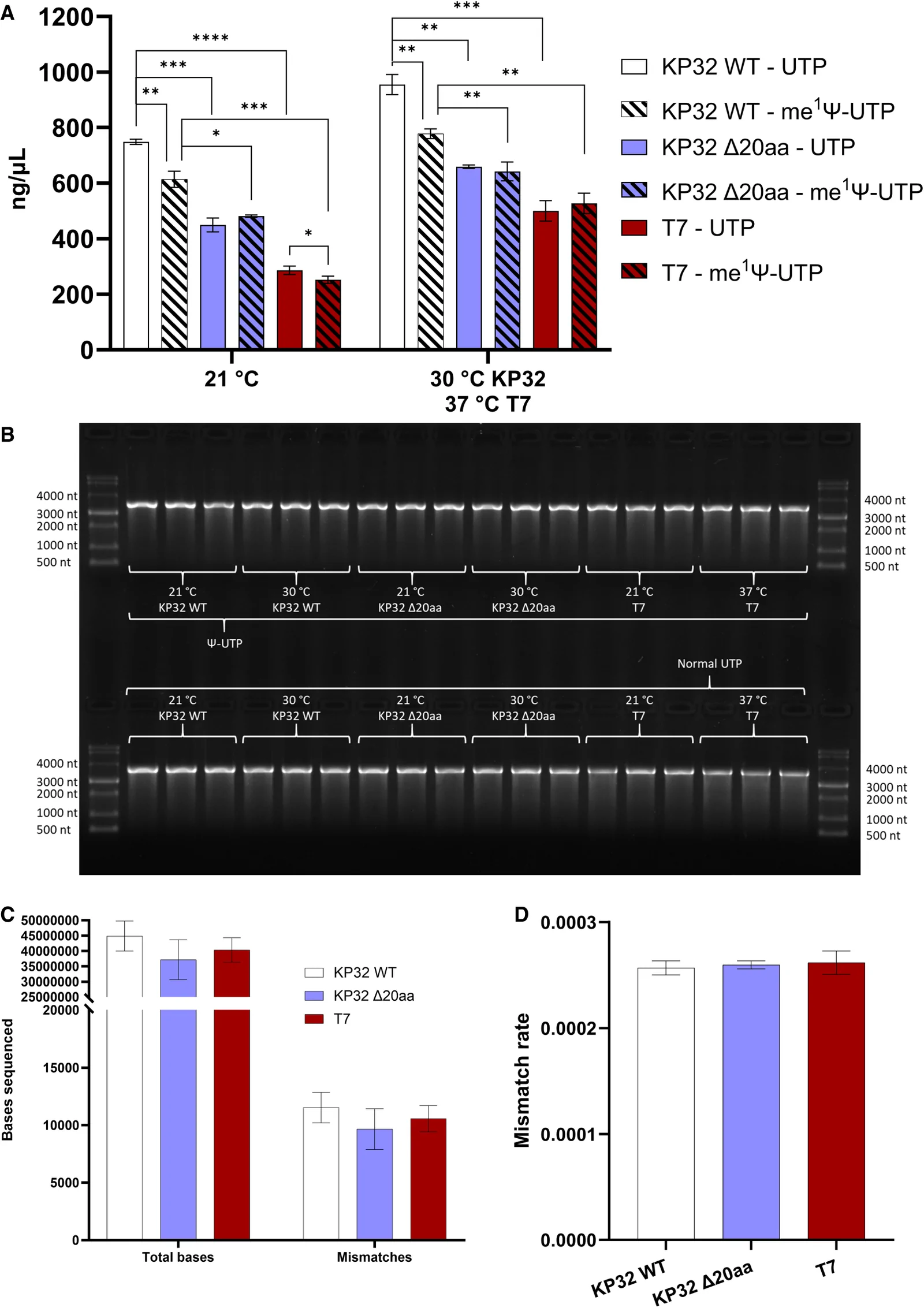
Analysis of incorporation of me^1^Ψ-UTP using the COVID-19 Pfizer-BioNTech mRNA vaccine sequence and RNAP fidelity assay. (*A*) Mass of RNA determined by nanodrop following incubation with rNTPs containing either standard UTP, or me^1^Ψ-UTP. Reactions were performed at either 21°C, or the optimum temperature for the RNAP utilized (30°C for KP32 WT and Δ20 aa RNAPs; 37°C for T7 RNAP). (*B*) Output of IVT reactions on 1% (w/v) agarose-TBE gel. Five hundred nanograms of total RNA for each reaction was loaded onto the gel. (*C*) Next generation sequencing results showing total number of bases analyzed and the number of mismatches detected in comparison to the SARS-Cov-2 mRNA reference sequence. (*D*) Comparative RNAP fidelity mismatch rate determined in this study. All reactions were performed in triplicate, and error bars represent the standard deviation. Where significance levels are indicated, unpaired *t*-tests with Welch's correction were performed. (*) *P* < 0.05, (**) *P* < 0.01, (***) *P* < 0.001, (****) *P* < 0.0001.

As a modified RNA nucleotide, me^1^Ψ-UTP was used within the Pfizer/BioNTech mRNA vaccine, comparison reactions were performed in the presence of this nucleotide, replacing unmodified UTP within the reaction. The results indicate that at optimum reaction temperatures, KP32 Δ20 aa and T7 RNAPs all used UTP and me^1^Ψ-UTP at statistically equal efficiencies, while KP32 WT had a preference for UTP (1.23-fold [*P* = 0.0054]). At 21°C there was a slight preference for UTP over me^1^Ψ-UTP in reactions with KP32 WT RNAP (1.22-fold [*P* = 0.0095]) and T7 RNAP (1.14-fold [*P* = 0.0414]). It was further found that KP32 WT RNAP produced a greater level of transcript than either KP32 Δ20 aa, or T7 RNAPs, when using me^1^Ψ-UTP at either 21°C (1.3- and 2.4-fold, respectively) or optimum reaction temperatures (1.2- and 1.5-fold, respectively). When 500 ng of RNA was loaded onto a 1% agarose-TBE gel (Fig. 5B), it was found that there was no clear difference between reactions using any of KP32 WT, KP32 Δ20 aa and T7 RNAPs, at any temperature, or when either unmodified UTP, or me^1^Ψ-UTP, was utilized.

The relative levels of dsRNA content were investigated by spot assay (Supplemental Fig. 5). This showed that dsRNA had been produced by all RNAPs tested here. However, it should be noted that dsRNA formation with this 4282 nt transcript could represent significantly more binding sites per molecule for the J2 dsRNA specific antibody than the 142 bp dsRNA positive control. As such, clear quantification of dsRNA could not be established, however it is clear that KP32 WT and Δ20 aa RNAPs produce approximately equal levels of dsRNA to T7 RNAP.

To investigate the fidelity of KP32 WT and KP32 Δ20 aa in comparison to T7 RNAP, ds-cDNA was generated from the purified mRNA vaccine produced with standard UTPs. A total of 500 bp was generated, representing the first 500 nt of the mRNA vaccine downstream from the TSS. Next generation sequencing was used to analyze individual transcripts, enabling the determination of mismatch rates in comparison to the reference sequence (Fig. 5C,D). The results show KP32 WT and Δ20 aa RNAPs have comparable levels of fidelity to T7 RNAP, previously reported as 5.3 × 10^−5^ error/base by Potapov et al. (2024). While our results indicate the T7 RNAP samples fidelity to the reference sequence was 2.6 × 10^−4^ error/base, it should be noted that this incorporates error rates for Superscript IV reverse transcriptase, and Q5 DNA polymerase. Our analysis was designed as a comparative analysis rather than to gain a definitive rate since this value was already known for T7 RNAP (Potapov et al. 2024).

### saRNA transcription to assess the benefit of the 20 aa motif in KP32 for long RNA transcript processivity

As a significant difference had been observed between KP32 WT and KP32 Δ20 aa RNAPs when analyzing the production levels of SARS-CoV-2 mRNA vaccine (Fig. 5), as well as the 1600 nt transcript (Fig. 4), it was hypothesized that the 20 aa motif in KP32 RNAP was beneficial for the processivity of transcripts. To further test this theory, the production of self-amplifying RNA (saRNA) was attempted utilizing a sequence developed during the COVID-19 pandemic as an alternative vaccination approach (McKay et al. 2020). These saRNA plasmids contained P_T7_ followed by the saRNA sequence consisting of the Trinidad donkey Venezuelan Equine Encephalitis Virus genome, with the SARS-CoV-2 spike protein gene replacing the viral structural proteins (McKay et al. 2020). The subsequent full length RNA transcript was 11,551 nt in length. saRNA require an exact sequence to function correctly intracellularly for the self-amplification of the payload RNA (Kulasegaran-Shylini et al. 2009; Hyde et al. 2015). The plasmid therefore by necessity utilized an AU initiating dinucleotide downstream from the RNAP promoter, rather than the GG utilized in other transcripts within this study. P_T7_ was swapped for P_KP32_, and a comparison assay for saRNA production levels was performed at optimum reaction temperatures with KP32, KP32 Δ20aa and T7 RNAPs (Fig. 6).

**FIGURE 6.**
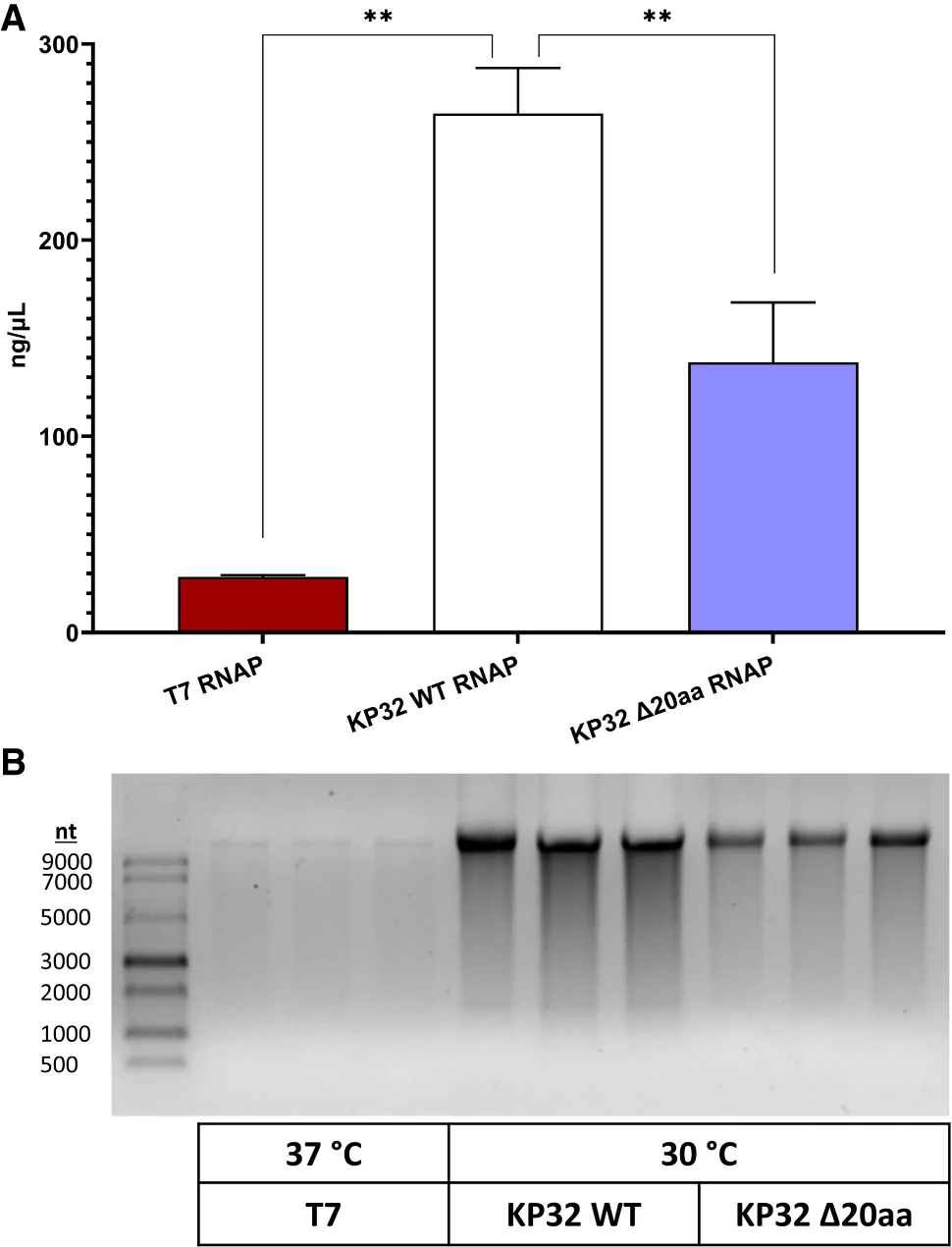
Analysis of saRNA long transcript processivity utilizing the native AU initiating dinucleotide. (*A*) Qubit readings for total RNA produced during saRNA IVT reactions at optimum reaction temperatures for KP32 WT, KP32 Δ20 aa and T7 RNAPs. (*B*) 1% agarose gel showing RNA products from triplicate saRNA IVT reactions. Where significance levels are indicated, unpaired *t*-tests with Welch's correction were performed. (**) *P* < 0.01.

The results show that KP32 WT RNAP outperformed both T7 RNAP (9.3-fold, *P* = 0.0032) and KP32 Δ20 aa (1.9-fold, *P* = 0.0057) for the production of saRNA with an AU initiating dinucleotide (Fig. 6A), and produced an RNA product of the intended length (Fig. 6B). This further indicates that the 20 aa motif retained in KP32 WT RNAP aids transcription processivity.

This result led to the construction and purification of a T7 RNAP variant containing the 20 aa motif from KP32 WT RNAP, to investigate if this 20 aa motif confers greater processivity for T7 RNAP. However, in both 1600 nt broccoli fluorescence assays (Supplemental Fig. 10A), and saRNA total RNA yield assays (Supplemental Fig. 10B), the T7 + 20 aa RNAP variant had a lower capacity for RNA production than the T7 WT RNAP re-purified alongside the new variant.

Significantly, T7 WT RNAP only produced 10.7% as much saRNA as KP32 WT RNAP. However, it should be emphasized that in this case it is likely that KP32 WT RNAPs increased output is likely to be largely due to initiation of transcription with an AU, rather than the usual GG used in other RNA transcripts within this study. This represents a clear improvement over the industry standard RNAP for saRNA production with the critical initiating AU dinucleotide.

Furthermore, the clear improvement in processivity when producing an RNA transcript initiating with an AU opens the utilization of KP32 WT RNAP to other transcripts where T7 RNAP struggles due to differing initiating dinucleotides. To investigate this further, IVT reactions were set up for each RNAP with the 1600 nt F30-broccoli template DNA containing the relevant promoter, and replacement of the usual GG initiating dinucleotide with every possible combination of nucleotides. These were tested in triplicate with reactions being run for 12 h. The results (Supplemental Figs. 11–14) show KP32 WT RNAP to have a clear increase in output rate, relative to T7 RNAP, across all templates with the exception of UU initiating dinucleotide which did not yield a clear increase in fluorescence for any RNAP. To compare KP32 WT and Δ20 aa RNAPs with T7 RNAP, Supplemental Table 3 was prepared where the 12 h fluorescence value is compared. This confirms a relative improvement in almost all conditions, with CA initiating dinucleotide in particular producing 812% more fluorescence from KP32 WT RNAP than T7 RNAP. AU initiating dinucleotide produced 174% greater RNA levels with KP32 WT RNAP than T7 RNAP, emphasizing the ability of KP32 WT RNAP to accept initiating dinucleotides otherwise hard to efficiently utilize.

## DISCUSSION

Here we have presented the RNAP from *Klebsiella* phage 32, exhibiting different characteristics to the current industry standard, T7 RNAP, with particular improvements in the production of saRNA transcripts with the native AU initiating dinucleotide. KP32 WT RNAP generated 9.3-fold higher levels of saRNA, in comparison to T7 RNAP. This significant increase in output was most likely due to an increased ability to utilize the native AU initiating dinucleotide, critical for the efficient intracellular production of the saRNA POI mRNA. Investigating this further, we show that KP32 WT RNAP outperforms T7 RNAP in production of our 1600 nt transcript when utilizing all possible combinations of initiating dinucleotide, with the exception of UU which did not yield efficient production from any RNAP. We consider this study to present KP32 WT RNAP as a strong candidate for the industrial production of saRNA therapeutics in the future in a cost-effective manner due to robust environmental tolerances, increased iPP tolerance, and effective utilization of modified RNA nucleotides in vitro.

We show that the KP32 bacteriophage genome holds at least three sequences which KP32 WT RNAP can recognize to initiate transcription and identified a strong promoter to go forward for IVT reactions. KP32 WT RNAP was found to be highly soluble within *E. coli*, leading to an efficient two step purification procedure, yielding pure RNAP. This purification approach uses the well-established T7 expression system within *E. coli*. However, KP32 WT RNAP-driven expression could be a feasible alternative to T7 RNAP in certain use cases as we have found it to have higher activity at lower temperatures than T7 RNAP in vitro. Furthermore, the utilization of both KP32 WT RNAP and T7 RNAP in tandem could enable new genetic circuits to be investigated. Alternatively, KP32 RNAPs relatively low-level acceptance of P_T7_ could be utilized in tandem with P_KP32_ where low and high mRNA production of different POIs are desired, for example in metabolic engineering strain construction where a protein is toxic at elevated expression levels.

We next sought to characterize the KP32 WT RNAP in vitro, as well as a variant of the polymerase where a 20 aa motif, not found in T7 RNAP, was removed from the N-terminal domain. Our results indicate this 20 aa motif, largely conserved within the *Klebsiella* phage family, improves the efficiency by which the RNAP produces mature RNA, in particular the SARS-CoV-2 mRNA vaccine and a 11,551 nt saRNA transcript. However, the exact mechanism by which this 20 aa motif improves polymerase performance is still to be investigated. We have found that both the KP32 WT and KP32 Δ20 aa variant RNAPs optimally transcribe RNA at 30°C, while reducing the temperature to 21°C maintains 71.4% and 70.2% of production, respectively (Fig. 3A). This high activity rate over a wider temperature range than T7 RNAP, as well as a broader pH and NaCl tolerances (Fig. 3D), opens the possibility of utilizing KP32 WT RNAP within cell free reactions, paper-based or transcription-based biosensors in the field, without the need for temperature-controlled incubation of the transcription reaction to yield an output in a timely manner (Jung et al. 2020). Lastly, comparative dsRNA and fidelity assays were carried out by spot assay and NGS, respectively. We show that KP32 WT and Δ20 aa RNAPs produce no more dsRNA or transcription errors than T7 RNAP, indicating potential to directly substitute KP32 RNAP, and its promoter sequence, within established methodologies for RNA production where fidelity and ssRNA purity is vital.

A significant use case for RNAPs following the success of the COVID-19 vaccines is mRNA vaccine production. Our results indicate KP32 WT RNAP could be a useful tool for the efficient large-scale production of therapeutic mRNA. An increased tolerance to iPP would mean less iPPase is required within the reaction, thus reducing production costs. These production cost savings could be further increased through the need for less RNAP to obtain the same amount of final mRNA. Of critical importance to mRNA therapies is the use of me^1^Ψ-UTP as this lowers the immune response to the mRNA, thereby increasing mRNA stability and increasing translation of the mRNA (Karikó et al. 2008; Andries et al. 2015). We have found that KP32 RNAP incorporates me^1^Ψ-UTP into mRNA products at an equal efficiency when compared to reactions using standard UTP, at optimum reaction temperatures. KP32 WT RNAP therefore has significant potential for real-world applications within biotechnology, particularly for efficient saRNA production.

## MATERIALS AND METHODS

### In silico T7-like RNAP selection, structural analysis and promoter identification

KP32 RNAP was identified following a BLAST search of the NCBI database against the protein sequence of T7 RNAP. Structural models were generated using the AlphaFold 3 model on the AlphaFold server (Abramson et al. 2024); the highest ranked model for predicted accuracy (model 0) was used for all downstream analysis. Reverse structure searches to find alignment targets were carried out on the Foldseek Search Server (van Kempen et al. 2024) using the .cif file for KP32 RNAP model 0. The predicted model for KP32 RNAP and alignment target structures were loaded into PyMOL version 3.1.5.1 for visualization and alignment. KP32 RNAP AlphaFold model was aligned against PDB structure 1QLN (Cheetham et al. 1999) with the inbuilt alignment function of PyMOL and with standard parameters. A secondary alignment was carried out with 0 cycles of atom rejection in order to determine a global RMSD score for alignment in addition to the refined RMSD score quoted as standard by PyMOL. To predict potential promoter regions specific for KP32 RNAP, the *Klebsiella* Phage 32 (KP32) genome (NC_013647.1) was screened by alignment of the intergenic regions with the 18 bp promoter sequence for T7 RNAP (5′-TAATACGACTCACTATAG).

### In vivo promoter validation

*E. coli* TOP10 cells were transformed with two plasmids where one, based on the pBAD33 plasmid, expressed KP32 RNAP, or T7 RNAP, under control of the arabinose inducible P_BAD_ promoter, utilizing the weak ribosome binding site (RBS) BioBrick part BBa_B0031. The second plasmid encodes GFP and is based on pET28a with *lacO* removed and the RBS replaced with BioBrick part BBa_BB0034. When analyzing putative KP32 RNAP promoter sequences, the T7 promoter (P_T7_) was also replaced. Overnight LB cultures supplemented with 1% glucose were spotted onto LB agar plates supplemented with 0.2% arabinose to induce expression of the RNAP. Plates were grown for 4 h at 37°C, and GFP expression was observed by analyzing for fluorescent *E. coli* colonies when exposed to blue light with an amber filter (470/530).

To assess fluorescence when cells were grown in liquid culture, transformants with both plasmids were grown overnight at 37°C with agitation in LB supplemented and 1% glucose. This culture was used to inoculate an expression culture of LB supplemented with 0.2% arabinose, and a repression culture of LB supplemented with 0.2% glucose. These were grown for 4 h at 37°C with agitation, after which OD (600 nm) and fluorescence (485/520) was measured with a FLUOstar Omega Microplate Reader.

### KP32 RNAP promoter transcriptional start site identification

KP32 promoter transcriptional start site (TSS) was identified using the New England Biolabs Template Switching RT Enzyme Mix. The manufacturer's instructions were followed, wherein a template switching oligo is utilized to add a barcode 3′ of cDNA generated from the RNA transcript. PCR amplification and Sanger sequencing of the cDNA then identifies this barcode and the adjacent transcriptional start site. Oligonucleotides used can be found in the Supplemental Material.

### Expression, purification and storage of KP32 WT, KP32 Δ20 aa and T7 RNAPs

To enable purification, KP32 WT RNAP and the KP32 Δ20 aa RNAP variant were cloned into pET28a with an N-terminally fused hexa-histidine tag, followed by a 10 aa sequence to aid protein solubility (SSGLVPRGSH) (Ishibashi et al. 2004). These plasmids were designated pMT002 (WT) and pMAP086 (Δ20 aa). These plasmids were used to transform *E. coli* BL21 Gold (DE3) cells. An overnight culture was used to inoculate a subsequent primary growth culture to OD_600_ 0.05 before growing to mid log phase at 37°C with agitation. Once at this stage of growth, the cultures were chilled to 18°C and expression of KP32 WT RNAP or KP32 Δ20 aa RNAP was induced with 0.1 mM IPTG. The cultures were grown with agitation at 18°C for 24 h before cells were harvested and pellets stored at −20°C.

T7 RNAP was modified to include a hexa-histidine and Tobacco Etch Virus (TEV) protease tag on the N-terminal within the pAR1219 plasmid (Davanloo et al. 1984), producing plasmid pMAP112. This was used to transform *E. coli* BL21 cells. An overnight culture was used to inoculate a subsequent primary growth culture to OD_600_ 0.05 before growing to mid log phase at 37°C with agitation. Cultures were induced with a final concentration of 0.1 mM IPTG and grown further at 37°C with agitation for 5 h before cells were harvested and pellets stored at −20°C.

To enable purification of each RNAP, amino acid sequences for which can be viewed in the Supplemental Material, cells were resuspended in lysis buffer (50 mM NaH_2_PO_4_, 300 mM NaCl, 10 mM imidazole, pH 8.0, supplemented with benzonase and cOmplete Protease Inhibitor Cocktail Tablet) and lysed with a Constant Systems Cell Disruptor at 25 kPsi. The cell debris was removed by centrifugation before 0.22 µm filtration of the supernatant. The lysate was loaded onto a 1 mL NiNTA column preequilibrated with the lysis buffer. The column was washed with buffer (50 mM NaH_2_PO_4_, 300 mM NaCl, 20 mM imidazole, pH8.0) before eluting in the same basic buffer on a gradient of 20–1000 mM imidazole over 20 column volumes. 0.5 mL fractions were collected during elution and analyzed by SDS-PAGE. The three 0.5 mL elution fractions containing the highest concentration of RNAP were loaded onto a HiLoad 16/60 Superdex 200 pg preparative size exclusion chromatography (SEC) column, preequilibrated with 2× RNAP storage buffer (100 mM Tris-HCl, 2 mM EDTA, 200 mM NaCl, pH 7.9). The protein was eluted with isocratic elution using the 2× RNAP storage buffer. One milliliter of elution fractions was collected, and the main peak was analyzed by SDS-PAGE to determine which fraction contained the RNAP at the highest purity.

Isolated RNAP protein fractions were diluted to 1× RNAP storage buffer by the addition of an equal volume of 100% glycerol, as well as 20 mM 2-mercaptoethanol and 0.1% (v/v) triton X-100. The final protein was stored at −20°C. Lastly, the total protein concentration was measured by Pierce 660nm Protein Assay Reagent.

### In vitro transcription activity assays

The fluorescent output of the broccoli RNA aptamer fused to an F30 scaffold, when in combination with the fluorophore DFHBI, enables side-by-side comparison of RNAP candidates at 25°C (Kartje et al. 2021). A 1600 bp GFP-Broccoli-F30 PCR product (the sequence and PCR primers for which can be found in the Supplemental Material) was used as DNA templates to assay different RNAPs, their characteristics and utilization of different initiating dinucleotides. Unless otherwise stated, fluorescence assays were performed in black, low volume 384 well plates in triplicate 12.5 µL reactions consisting of 1× RNAP reaction buffer (40 mM Tris.HCl, 6 mM MgCl_2_, 1 mM DTT, 2 mM spermidine, pH 7.9), 8 µM DFHBI, 1 mM rNTPs (sodium salt), 4 U/μL RiboLock RNase Inhibitor, 0.1 unit of inorganic pyrophosphatase, 10 mM KCl, 125 fmol template DNA and 11.25 nM RNAP. Reactions were analyzed with a FLUOstar Omega Microplate Reader at 485/520 nm, gain 1692, at 25°C.

To analyze RNA production without fluorescence, reactions were set up with the same reaction mix as for fluorescence assays, without the addition of DFHBI. Reactions were quenched by addition of RQ1 DNase and RQ1 DNase buffer and incubation at 37°C for 15 min. The RNA within the reactions was then purified using Monarch RNA Cleanup Kit, and elution in H_2_O at the same volume as the initial IVT reaction. Following quantification, total RNA concentration was normalized, and an equal volume of purified RNA was mixed with 2× RNA loading dye (NEB). Samples were heated to 70°C for 5 min, before chilling to 4°C for 1 min. Five hundred nanograms of total RNA was loaded immediately onto a 1% agarose-TBE gel with 1× SafeView nucleic acid stain added. Alongside samples, ssRNA Ladder (NEB) was added, and the gel was run at 100 V for 30 min. Gels were analyzed with Bio-Rad Gel Doc Imaging System. When reactions testing the utilization of me^1^Ψ-UTP were performed, the modified nucleotide was used to replace UTP in the rNTP stock, maintaining the same concentration.

### SARS-CoV-2 mRNA vaccine production assay

To investigate the comparative rate of SARS-CoV-2 mRNA vaccine production, plasmids were constructed from synthesized DNA and P_KP32_ or P_T7_ was inserted upstream of the gene encoding the spike protein, producing plasmids pMAP164 and pMAP165, respectively. MluI linearized plasmids were prepared as IVT reaction templates. IVT reactions totaling 40 µL were prepared. These consisted of 1× RNAP reaction buffer, 1 mM rNTPs (sodium salt) or me^1^Ψ-UTP (sodium salt), 4 U/μL RiboLock RNase Inhibitor, 0.1 unit of inorganic pyrophosphatase, 100 fmol template DNA and 16 nM RNAP. Reactions were quenched with RQ1 DNase treatment. The RNA was purified using Monarch RNA Cleanup Kit, eluting in H_2_O at the same volume as the initial IVT reaction. RNA mass was quantified by nanodrop analysis. The sequence of the IVT template DNA can be found in the Supplemental Material.

### saRNA production analysis

KP32 RNAP specific plasmids were created by modification of the previously reported COVID-19 nCoV—Clean Cap VEEV plasmid (McKay et al. 2020). This produced plasmid pMAP096 where P_T7_ had been replaced with P_KP32_. Importantly, these promoters removed the standard TSS for each RNAP, utilizing the A initiating nucleotide native to the VEEV. MluI linearized plasmids were prepared as IVT reaction templates. To test production of saRNA transcripts, IVT reactions totaling 40 µL were prepared. These consisted of 1× RNAP reaction buffer, 1 mM rNTPs (sodium salt), 4 U/μL RiboLock RNase Inhibitor, 0.1 unit of inorganic pyrophosphatase, 100 fmol template DNA and 150 nM RNAP. Reactions were quenched with RQ1 DNase treatment. The RNA was purified using Monarch RNA Cleanup Kit, eluting in H_2_O at the same volume as the initial IVT reaction. RNA mass was quantified using the qubit RNA broad range assay. The sequence of the IVT template DNA can be found in the Supplemental Material.

### RNAP fidelity analysis

Three micrograms of purified RNA from IVT reactions producing the SARS-CoV-2 mRNA vaccine with standard rNTPs were used in reverse transcription reactions using Superscript IV. A specific oligo(5′-GTTGTTCTTGTGGTAGTAGACG) was used to generate a 500 nt ss-cDNA product representing the first 500 nt of the vaccine sequence. RNA, dNTPs and the oligo were preincubated together at 65°C for 5 min and 4°C for 2 min. Subsequently, dithiothreitol, RiboLock and Superscript IV reverse transcriptase buffer and enzyme were added and incubated at 55°C for 20 min and 80°C for 10 min. RNase 1 was added directly and incubated at room temperature for 30 min to remove the RNA. Second strand synthesis was performed by further addition of another oligo representing the TSS and downstream nucleotides of the vaccine sequence (5′-GGAATAAACTAGTATTCTTCTGG), water, and Q5 DNA polymerase buffer and enzyme. This was incubated at 98°C for 30 sec, 58°C for 30 sec and 72°C for 2 min. The product was first PCR purified, then run on a 1% agarose gel for excision and purification of the main product at 500 bp. To enable sequencing, a 17-cycle PCR reaction using Q5 DNA polymerase and the same oligos as used in ds-cDNA preparation above was performed. The reaction was PCR purified and checked by agarose gel to ensure the 500 bp target was the only detectable product. The product was sent for Amplicon-EZ next generation sequencing (NGS) with Genewiz. Sequencing was performed with the Illumina platform to produce 251 bp paired end reads.

Raw reads were trimmed to remove low-quality bases and standard Illumina adapters using “cutadapt” (Martin 2011) (version 4.6) in paired end mode -a AGATCGGAAGAG -A AGATCGGAAGAG -m 20 -q 30. Reads were further trimmed to remove primers with the parameters -a CGTCTACTACCACAAGAACAAC -A CGTCTACTACCACAAGAACAAC -a CCAGAAGAATACTAGTTTATTCC -A CCAGAAGAATACTAGTTTATTCC -g GTTGTTCTTGTGGTAGTAGACG -G GTTGTTCTTGTGGTAGTAGACG -g GGAATAAACTAGTATTCTTCTGG -G GGAATAAACTAGTATTCTTCTGG -m 20 -q 30.

Trimmed reads were aligned to the template sequence using “bwa mem” (Li and Durbin 2010) (version 0.7.18), and the resulting SAM files were converted into coordinate sorted and indexed BAM files using “samtools” (Li et al. 2009) (version 1.22.1). Alignments were converted into “mpileup” format using “bcftools mpileup” (Danecek et al. 2021) (version 1.20) with the parameters “-Q 30 -q 20 -a AD, DP ‐‐max-depth 1,000,000”, then converted into VCF files using “bcftools call” with the parameters “-Am.” The number of reads supporting the alternative allele and supporting the reference allele at each position was extracting into a text file using the “GATK” (Van der Auwera et al. 2013) (version 4.1.6) tool “VariantsToTable”, with the parameter “-F DP4.” For each sample, the total number of aligned bases was calculated as the total number of reads supporting the reference or the alternative allele across all sites, and the total number of mismatching bases was calculated as the total number of reads supporting the alternative allele across all sites. The rate of mismatch between sequence and reference was calculated as mismatching bases/total bases.

### dsRNA spot assay

To investigate the relative level of dsRNA production between the RNAPs, a spot assay was performed. The total RNA concentration of purified IVT reaction products was normalized, and equal volumes, totaling 1000 ng RNA, were spotted on a positively charged nylon membrane. Equal volumes of 142 bp dsRNA (Jena Bioscience) positive control and poly(A) negative control were also spotted. Dilutions of the dsRNA control was also spotted while retaining equal volumes loaded to retain potential RNA diffusion levels on the membrane. All spots were allowed to dry at room temperature before blocking the membrane with 5% (w/v) skimmed milk powder in TBS-T (50 mM Tris, 150 mM NaCl, 0.05% [v/v] Tween-20, pH 7.4) for 1 h with agitation at room temperature. The membrane was next incubated overnight at 4°C with agitation with 1% (w/v) skimmed milk powder in TBS-T and anti-dsRNA J2 antibody produced in mouse (Merck) at a 1 in 1428 dilution. The membrane was washed three times with TBS-T before incubation for 1 h with agitation at room temperature with 1% (w/v) skimmed milk powder in TBS-T and anti-mouse IgG-peroxidase antibody produced in rabbit (Merck) at a 1 in 10,000 dilution. The bound antibody was visualized with 0.1 mL/cm^2^ of WesternSure Premium Chemiluminescent Substrate (LICORbio) for 5 min at room temperature with agitation, before imaging with a LICORbio C-Digit.

## DATA DEPOSITION

The data underlying this article are available in Figshare, at https://figshare.com/s/25b3add597bab2c32c64.

## SUPPLEMENTAL MATERIAL

Supplemental material is available for this article.

## Figures and Tables

**Marcus A. Price:**
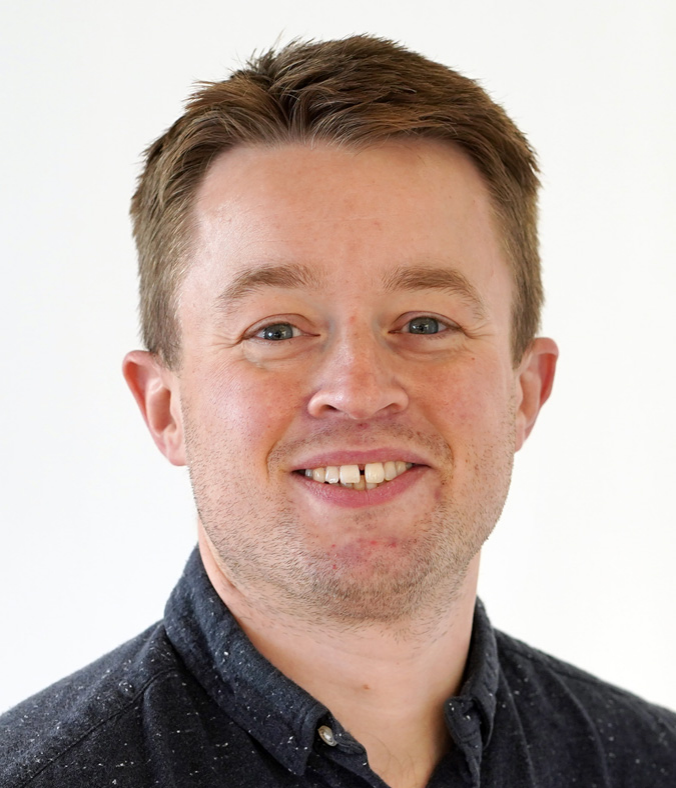


## MEET THE FIRST AUTHOR

***Meet the First Author(s)* is an editorial feature within *RNA*, in which the first author(s) of research-based papers in each issue have the opportunity to introduce themselves and their work to readers of *RNA* and the RNA research community. Marcus A. Price is the first author of this paper, “Efficient production of a self-amplifying RNA vaccine sequence with the single subunit RNA polymerase from *Klebsiella* phage 32 utilizing the native AU initiating dinucleotide.” Marcus is a postdoc within the lab of Professor Susan Rosser, in the School of Biological Sciences at The University of Edinburgh. The lab focuses on control systems for cell and gene therapies, including RNA tools, and Marcus is currently focusing on in vitro RNA production methodologies, as well as developing novel RNA therapeutics.**

**What are the major results described in your paper and how do they impact this branch of the field?**

Our paper characterizes the RNA polymerase (RNAP) from *Klebsiella* phage 32. We found KP32 RNAP to produce up to 9.3-fold more self-amplifying RNA when utilizing the native and functionally critical AU initiating dinucleotide, in comparison to T7 RNAP. Additionally, we investigated tolerance to all differing initiating dinucleotide variants, which, on all templates except that starting with UU, revealed KP32 RNAPs increased ability to generate transcripts. Further, comparing to T7 RNAP throughout, we investigated KP32 RNAP optimal reaction conditions, as well as tolerances to other factors, such as NaCl.

These results reveal KP32 RNAP to be a useful addition to the RNAP toolkit for use within in vitro RNA transcription at lab or commercial scale. While there is a clear particular focus on saRNA transcription, increased tolerance to environmental factors such as NaCl could be of interest to those investigating paper-based biosensors, and orthogonality enables the use of KP32 RNAP alongside T7 RNAP within gene circuits.

**What led you to study RNA or this aspect of RNA science?**

Initially this project started as we perceived there to be a need for more tools for gene circuits and transcriptional control mechanisms within synthetic biology. However, with the COVID-19 pandemic there came more focus on RNA production methodologies, not least with the tremendous success of the mRNA vaccines.

More broadly, I believe RNA control mechanisms and tools have a lot of potential to transform healthcare with the next generation of therapies. I find working toward these objectives to be really exciting, and a real driver in my career. Moreover, my father was fortunate enough to recently receive CAR-T therapy to successfully treat and cure his B cell lymphoma. Having such a close experience to the power of cutting-edge healthcare has made me even more keen to contribute, where I can, to develop these treatments further.

**What are some of the landmark moments that provoked your interest in science or your development as a scientist?**

As a child I remember learning about the cloning of Dolly the sheep in Edinburgh. This really caught my imagination as to what could be possible in the near future, and when I decided to go to university, Edinburgh was the clear choice. When I started working after my undergrad, I had a very positive early experience of being pushed to explain my work and results. This was invaluable and made me ensure I was always well prepared for future meetings. During my PhD, I had the very exciting opportunity to present my work on CRISPR-based genome editing of *Bacillus subtilis*, directly after Emmanuelle Charpentier presented on the discovery of CRISPR. Needless to say, this was nerve-wracking but another very valuable experience to a high-pressure situation. At the end of my PhD, I also had a really positive viva experience, with the meeting becoming a nice conversation about my work in general. It was a really pleasant surprise, and one for which I'm very thankful. Lastly, and probably most importantly, I'm thankful to have started my career in an environment encouraging a strong work-life balance ethos. I feel this has set me up well for the long run and it's something I try to emphasize to new students as they enter the workplace.

**If you were able to give one piece of advice to your younger self, what would that be?**

In Scotland it is very possible to go to university at the age of 16. I went when I was 17, however I still look back and think that was too young. I didn't get as much as I could, academically, out of my undergraduate. If I could go back to my teenage self, I would say there is nothing wrong with leaving school at 17, but before going onto tertiary education go and see the world and get some life experience. This is something I did between my undergrad and starting my PhD when I worked for 3.5 years in industry. It meant I got so much more out of my PhD than I think I would have if I'd gone directly into one, including a 3-month placement at a biotechnology research institute in Tianjin, China—an incredible experience!
